# Semiactive Knee Orthotic Using a MR Damper and a Smart Insole to Control the Damping Force Sensing the Plantar Pressure

**DOI:** 10.3389/fnbot.2022.790020

**Published:** 2022-05-30

**Authors:** David Alvarado-Rivera, Paola A. Niño-Suárez, Leonel G. Corona-Ramírez

**Affiliations:** ^1^Instituto Politécnico Nacional, Escuela Superior de Ingeniería Mecánica y Eléctrica, México City, Mexico; ^2^Instituto Politécnico Nacional, Unidad Profesional Interdisciplinaria en Ingeniería y Tecnologías Avanzadas, México City, Mexico

**Keywords:** knee orthotic, MR damper, smart insole, PD control, GRF

## Abstract

This work presents the development of semiactive knee orthosis prototype that focus to absorb the forces and impacts in this joint during the human gait. This prototype consists of three subsystems: the first is a wireless and portable system capable of measuring the ground reaction forces in the stance phase of the gait cycle, by means of an instrumented insole with force sensing resistors strategically placed on the sole of the foot, an electronic device allows processing and transmit this information via Bluetooth to the control system. The second is a semiactive actuator, which has inside a magnetorheological fluid, highlighting its ability to modify its damping force depending on the intensity of the magnetic field that circulates through the MR fluid. It is regulated by a Proportional Derivative (PD) controller system according to the values of plantar pressure measured by the insole. The third component is a mechanical structure manufactured by 3D printing, which adapts to the morphology of the human leg. This exoskeleton is designed to support the forces on the knee controlling the action of the magnetorheological actuator by ground reaction forces. The purpose of this assistance system is to reduce the forces applied to the knee during the gait cycle, providing support and stability to this joint. The obtained experimental results indicate that the device fulfills the function by reducing 12 % of the impact forces on the user's knee.

## Introduction

Knee injuries are one of the most common problems in the population, mainly injuries in soft tissues such as ligaments, these can suffer partial or total ruptures. Then, it is necessary to use systems that can offer support and stabilization in this joint during rehabilitation process, the most common are orthoses.

A knee orthosis is an orthopedic device exerting a therapeutic or preventive effect on this joint, reducing, or rehabilitating injuries to the ligaments, mainly caused by trauma that represents a disruption in the balance and mobility of the individual who suffers them (Nordin and Frankel, 2013). The development of orthotic devices has had an important growth in recent years, incorporating new elements that allow the user to recover in less time, in addition to offering a better adaptation to the movements when walking. The semiactive lower limb orthoses (SALLO) are one of the more studied options, this type of device is obtained by incorporating elements such as a magneto-rheological (MR) actuator to control the movement of the orthotic device and provide support to the knee joint during flexion and extension movements.

Based on the principles of mechatronics, where mechanical, electrical, and computational systems are integrated to develop a semi-active lower limb orthosis (SALLO), this system was developed which considers sensorimotor function. The objective of the SALLO presented in this article is reduce the forces on the knee is subjected during the gait cycle, controlling the damping force of a MR actuator, providing support to the structure coupled to the knee. The damping force is controlled by regulating the magnetic field flowing through the MR fluid, setting a relation between the input current and the ground reaction forces (GRF) measured by the instrumented insole.

The results obtained by carrying out different experiments with a healthy user to evaluate the efficiency of the insole during the gait cycle, indicated that the data acquired can represent the ground reaction forces since these information present changes depending on the position of the foot and contact with the ground. Similarly, the function of the orthosis is giving support of the knee joint, regulating the forces that it must withstand during rehabilitation processes or as an assistive device in the lower limb when the individual suffers knee injuries, was verified by using the orthosis with healthy subjects. At this time, the medical protocol required to perform experiments on subjects suffering from specific injuries to the knee joint is being prepared.

Section Overview of Related Work from this article presents the state of the art in the development of semi-active orthotics and GRF measurement insoles; Section Materials and Methods describes the stage design of the developed orthosis; In the Results Section, the data obtained from the experiments carried out at each of the stages of the system and the integrated system are analyzed. And in the Conclusions Section the benefit of incorporating MR actuators in the design of orthotic devices and the importance of acquiring GRF signals and using them for the control of the orthosis operation, considering the sensorimotor effects in the design, implementation, control, and use of portable robotic devices is discussed.

## Overview of Related Work

The development of prototype knee orthoses has been aimed at reducing the stresses to which this lower limb joint is subjected. To this end, in recent years various investigations have proposed using additional devices to conventional orthoses to limit the forces that interact in the knee, such as MR actuators, in addition to incorporating pressure sensors to measure GRF and thus controlling the damping force affecting the knee.

One of the first works reporting the development of a LLO is presented in Zite et al. (2006), where an MR damper is incorporated into the structure of the orthosis, this device was selected for its response speed, weight, and their relatively low energy consumption. According to the results, the authors indicate the benefits and ease use of an MR actuator for controllable orthosis design are remarkable. In Chen and Liao (2006), an exoskeleton is proposed using an MR actuator with a controllable torque, which acts as a brake or clutch according to the working states defined by the knee flexion angle and the ground reaction forces, using these data the actuator on the exoskeleton becomes more energy efficient. In Bulea et al. (2012), a mechanism was developed to assist people who have suffered some paralysis in the lower limb, which integrates an MR actuator that regulates the flexion movement of the knee from a variable impedance control technique, which incorporates a mechanism of four bars providing the torques required to perform leg movements, the total weight of the orthosis is high, about 3.5 kg. The MR actuator used is LORD RD-8040-1, from which the characteristic curve was obtained, determining that the increase in current flow increases the damping force of the actuator. The mechanism was tested with injured people, showing that the proposed system serves as a brake against knee flexion movements, and allows knee movements under high loads. In the work developed by Guo and Liao (2009), the design of a knee brace is presented to support the mobility of people with motor limitations, its main function is to reduce the movement of the knee during the gait cycle, by controlling the torque on the mechanism from the configuration of the actuator MR. The results mentioned by the authors indicate that the MR actuator supports the torque generated on the knee during the gait cycle.

On the other hand, instrumented insoles have been developed for the measurement of ground reaction forces or GRF. These systems collect information on the pressures generated in the sole of the foot during the gait cycle. In the work published by Ngueleu et al. (2019), FSR sensors distributed over the sole of the foot are used to accurately count the user's steps. The advantage of this type of system is the portability and the ability to generate an analysis of the gait cycle, as in the work developed by Tabrizi et al. (2018), wherein the development of an intelligent insole incorporating FSR sensors, a Bluetooth module, a battery, an accelerometer, as well as a microprocessor, to detect force peaks depending on the area of the foot that makes contact with the ground. In a similar way in Fang et al. (2017), an insole composed of eight FSR sensors distributed mainly in the forefoot, midfoot, and heel is presented, with this configuration a graph relating the force with which the sole of the foot contacts the ground is obtained.

According to the state of the art, developing a semiactive orthotic system to reduce the forces on the knee is a viable option to bring support in this joint during walking and other daily activities. However, few studies consider the sensorimotor effects of the joint and the musculoskeletal system associated with the lower limb in the design, implementation, control, and use of these automated orthotic devices.

In this proposal, by integrating a magneto-rheological actuator to the orthosis to regulate the damping force, avoiding that the knee suffers an overstrain when it is not able to support the weight of the user, and incorporating a system to measure the GFR during the gait cycle to evaluate the data necessary to control the response of the MR damper, a SALLO was obtained that self-regulates its damping according to the phase of the user's gait cycle and their physical-motor conditions.

## Materials and Methods

One of the main daily activities in humans is walking, due to its complexity, specific characteristics of the activity carried out by the extremities of the body have been identified during the gait cycle. The gait cycle is mainly divided into two phases, the stance phase, and the swing phase. During the stance phase, the contact of the foot with the ground is contemplated, while this action is carried out the leg and in particular the knee is subjected to high forces, according to (Meireles et al., 2017) these forces reach a value three times greater than the weight of a person. In the swing phase GRF is not recorded.

The knee is the most complex joint that exists in the human body, it is a biarticular structure composed of the tibiofemoral joint and the patellofemoral joint. In addition, it is one of the joints performing the most work during the locomotor activities, such as walking, sitting, or getting up. Injuries to the knee ligaments cause joint pain and instability, causing the loading forces on the knee increase, causing decompensation during the gait cycle. These injuries are related to sudden movements during some physical activity, such as running, changing direction quickly, in the fall after a jump or a collision directly on the knee. Depending on the degree of the injury is the time taken for recovery, the recovery time from an injury to these tissues can range from 2 weeks and extend to 66 months. To recover from this injury, the support of therapeutic systems such as orthoses is necessary.

**Figure 1:**
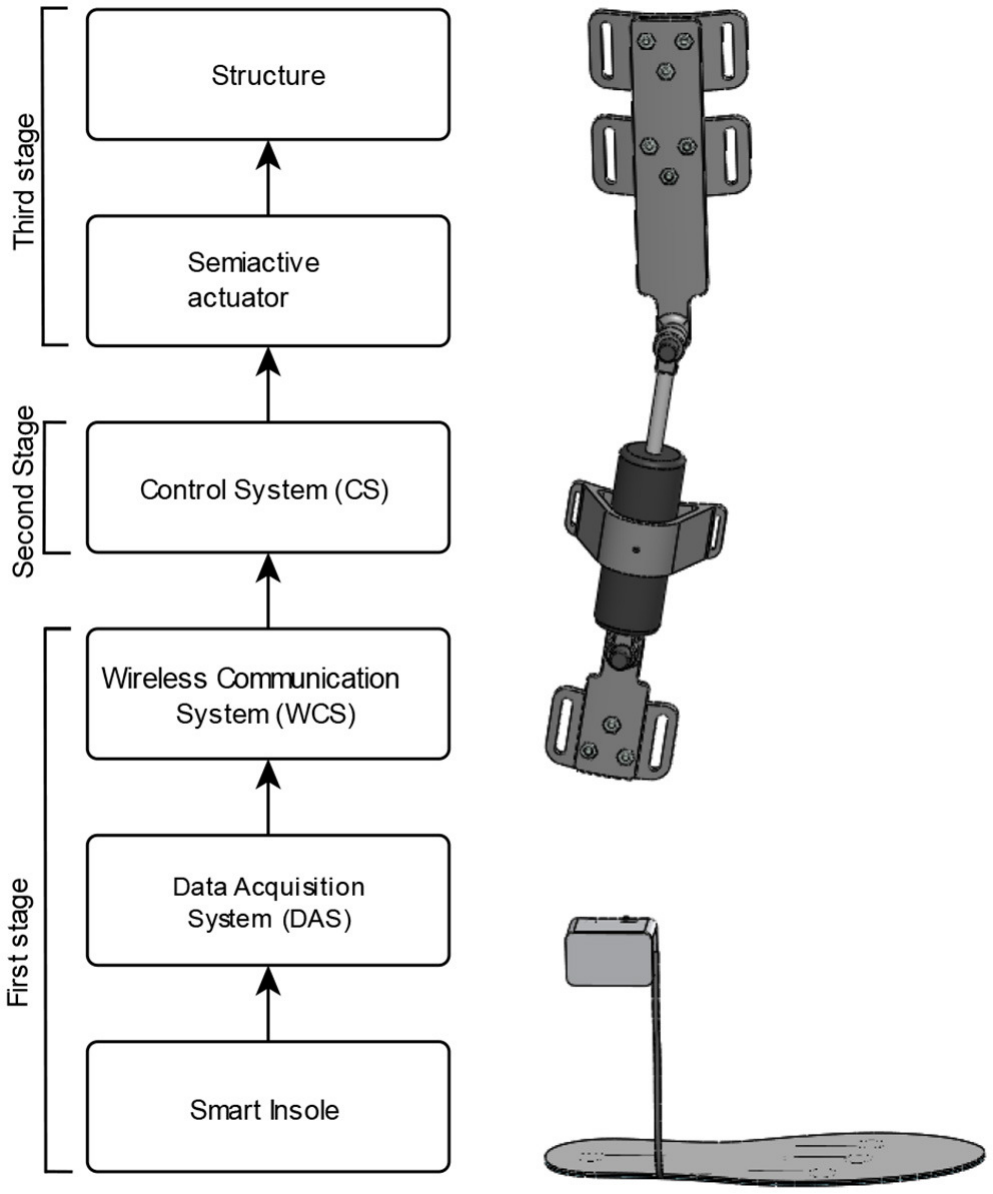
Composition of the orthosis using a semi-active actuator.

The orthosis system presented in this work aims to reduce the forces to which the knee is subjected during the gait cycle. For its design, the methodology “V Model,” presented in Forsberg and Mooz (1992) was used. As a result of the proposed methodology, in Figure 1, the stages that make up the developed orthosis are observed, the first stage is the instrumented insole with its data acquisition system that is responsible for obtaining, sending and processing the GRF generated in the sole of the foot of the user during the gait cycle; the second is the MR actuator control system and finally the structure that is operated by the MR actuator is observed and supports the knee joint, engaging the user's leg. The GRF obtained vary with respect to the gait cycle, this due to the movements of the lower limb, the control system uses these characteristics to vary the input current that reaches the MR actuator. In this way, when the GRF increases, the damping force of the actuator also increases, that means, the damping values of the system adapt according to the user's cadence, allowing control of the forces to which the knee is subjected, during different motor activities.

In the next sections will be described each stage of the SALLO developed as shown in Figure 1.

### Insole for GFR Measurement

**Figure 2:**
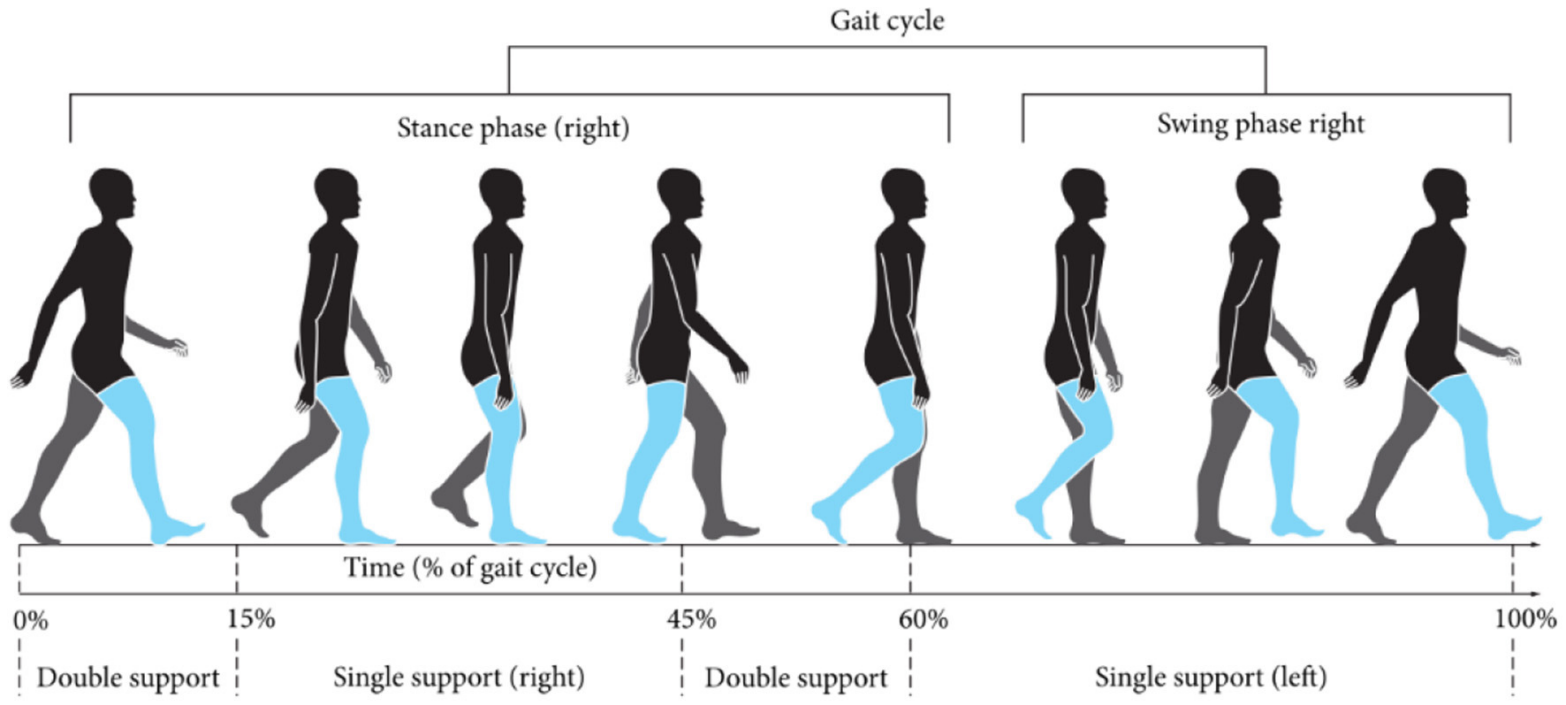
Human gait cycle (Cheze et al., 2019).

When analyzing the human gait cycle, it is observed that the stance phase lasts for ~60% of the total cycle, while the remaining 40% corresponds to the take-off phase (Perry, 2010). Each phase in turn is subdivided into shorter periods, according to the movements made by each leg when taking a step. In Figure 2, the contact points of the foot change during the gait cycle, as does the value of the pressure exerted by each part of the foot on the ground, is observed (Muñoz-Organero et al., 2017). To measure these GFRs during the gait cycle, a flexible and lightweight instrumented insole was developed, made of silicone, which is placed inside the user's footwear. Inside the insole, four resistive force sensors, FSR, were located to measure the variation of force that exists in different areas of the foot during the gait cycle.

**Figure 3:**
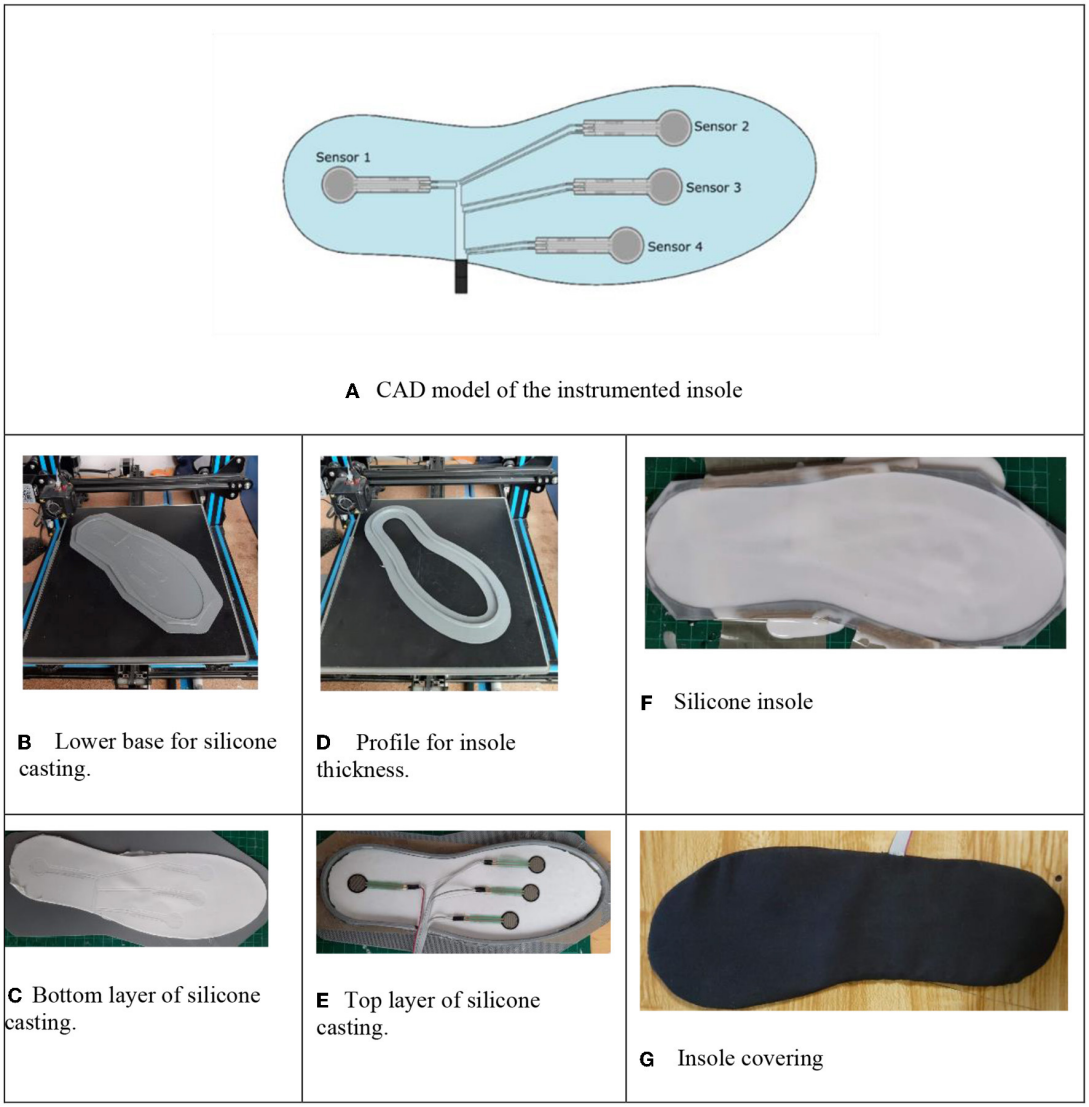
Instrumented insole, CAD design, and manufacturing. **(A)** CAD model of the instrumented insole. **(B)** Lower base for silicone casting. **(C)** Bottom layer of silicone casting. **(D)** Profile for insole thickness. **(E)** Top layer of silicone casting. **(F)** Silicone insole. **(G**) Insole covering.

The sensor distribution on the insole were defined from the foot areas wherein the larger part of the GFR during the gait cycle are concentrated according to Muñoz-Organero et al. (2017), as observed in Figure 3A. The first contact is made on the heel, depending on the cadence when walking, the rest of the foot is gradually placed on the ground. Before starting the swing phase, it is on the tip of the foot where most of the GFRs are concentrated. From this analysis, the distribution of FSR sensors in the insole was made, as seen in the CAD model made in SolidWorks^®^ 2016, presented in Figure 3A. FSR sensors vary their resistive value because a pressure on their contact area (Duarte, 2018) which is why these were selected for the development of the insole.

The fabrication of the insole follows the next process; first the deposition of the bottom layer, a base was printed by additive manufacturing that allowed to identify the position of the sensors, showed in Figure 3B. The first layer of silicone was poured, in a ratio of 60 g of silicone to 3 g of coagulant in Figure 3C is observed how the position of the sensors were marked on the silicone. A profile with a thickness of 5 mm was printed by additive manufacturing to contain the cast of the remaining silicone in Figure 3D, over the first layer of the insole and the sensor array are placed on the profile (Figure 3E). The sensors are covered with a second layer of 60 g of silicone, reaching the desired thickness (Figure 3F). The casting is carried out gradually to avoid the formation of air bubbles, which would cause errors in the reading of the sensors. Finally, a layer of cotton cloth is placed over the silicone, to give the appearance of a common insole, greater comfort, and ergonomics to the user (Figure 3G).

#### Sensors for GFR Measurement

**Figure 4:**
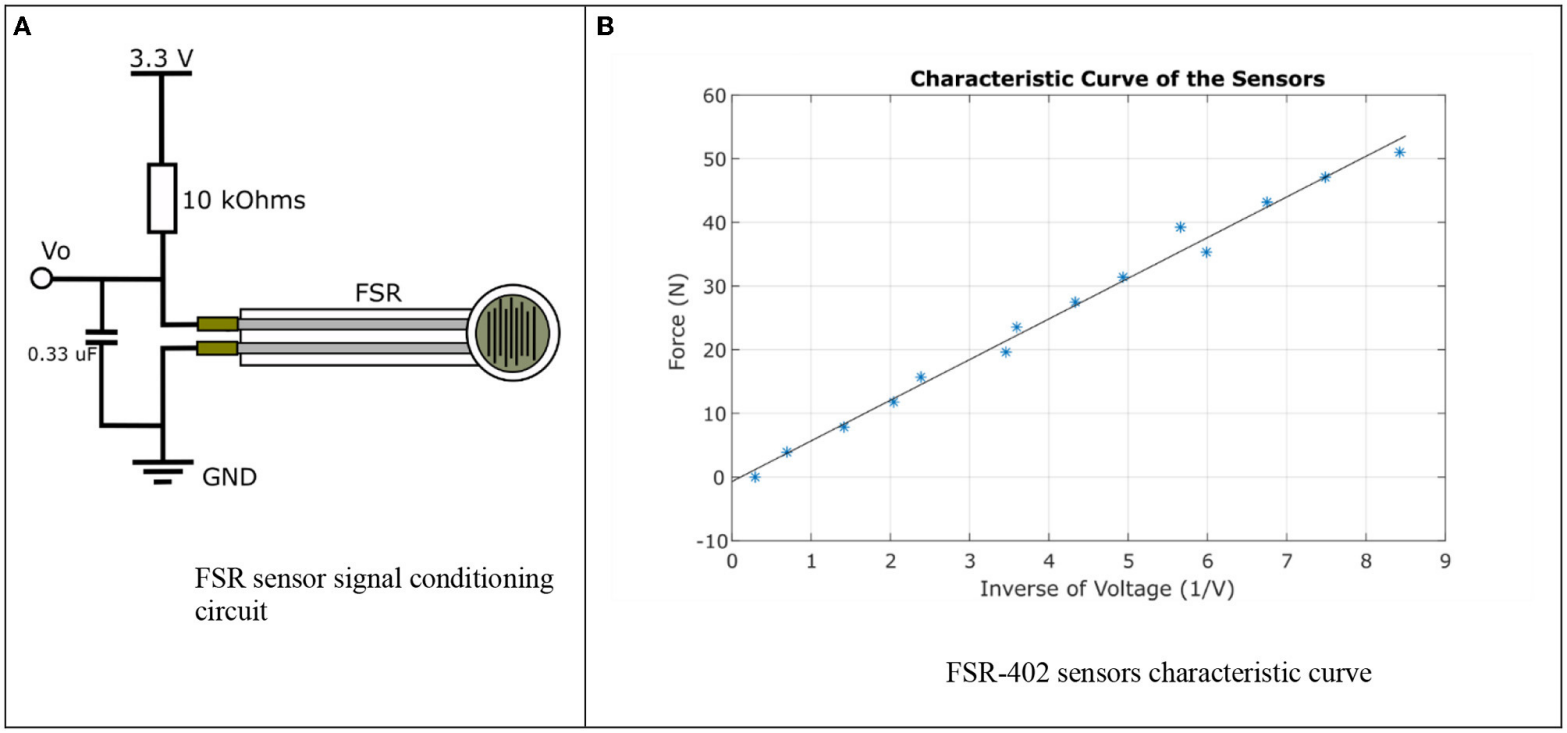
FSR sensors characterization. **(A)** FSR sensor signal conditioning circuit. **(B)** FSR-402 sensors characteristic curve.

Force sensitive resistor FSR 402 were selected for their size, operating range, and ease of implementation. According to its technical specifications, the sensor exhibits an approximately linear behavior when referring to its conductance. To characterize the sensor, an experiment was designed, where an FSR sensor was covered with silicone, a load was collocated on the circular area, this weight varied from 0.4 to 5.4 Kg, with 0.4 Kg increments. For each mass, 30 sensor readings were obtained through the output voltage of the conditioning stage observed in Figure 4A. The voltage divider implemented as a signal conditioning system allowed the sensor to reach a sensitivity of 0.8 mV/N. Additionally, an analog low pass filter was incorporated to eliminate white noise caused by the electrical system.

The statistical analysis of the data obtained was performed and the average behavior of the sensor was plotted in Figure 4B, where the horizontal axis corresponds to the inverse of the voltage and the vertical axis to the applied force.

From the graph in Figure 4B, an approximately linear mathematical model of the sensor was obtained, a first order polynomial presented in (1), where *F* is the resultant force to apply a load on the sensor and *V*_*o*_ is the inverse of the voltage measured.


(1) $$\begin{array}{r} F=6.3841V_{o}-0.7007 \end{array}$$

#### Data Acquisition System

**Figure 5:**
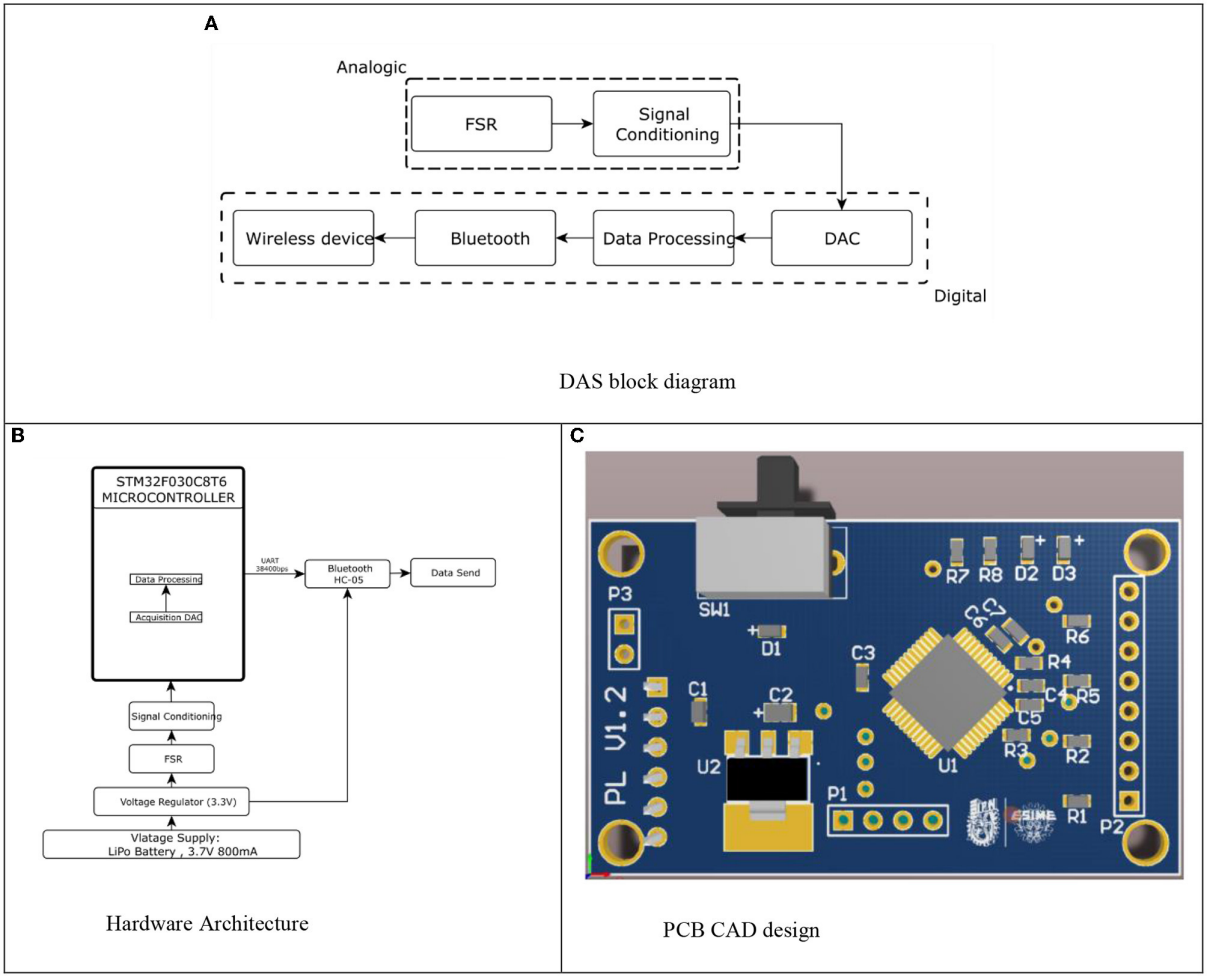
Data acquisition system. **(A)** DAS block diagram. **(B)** Hardware Architecture. **(C)** PCB CAD design.

A data acquisition system (DAS) was designed to send the information acquired by the sensors to the MR actuator control stage, its block diagram is presented in Figure 5A. In Figures 5B,C, the hardware architecture of the DAS and the PCB card that designed to integrate surface mount device SMD, to obtain a reduced-size DAS module, is presented.

The data obtained by the DAS is sent via Bluetooth to any digital device storing it in a text document and can be graphed using a mobile application.

#### Instrumented Insole Validation

**Figure 6:**
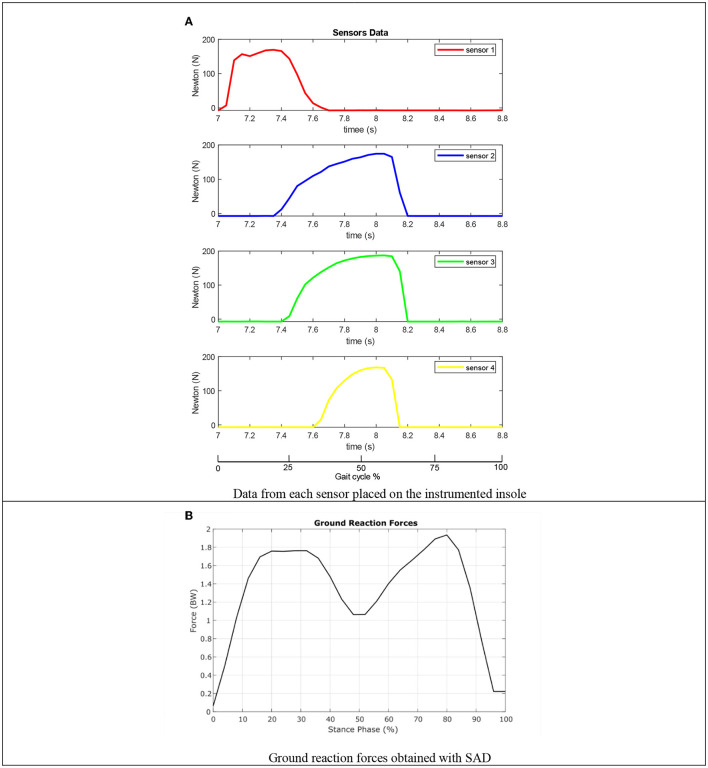
Data processed using the DAS. **(A)** Data from each sensor placed on the instrumented insole. **(B)** Ground reaction forces obtained with SAD.

Experiments were carried out to verify and validate the operation of the insole with a healthy user 1.8 m tall and 80 kg weight, in a closed room with a temperature of 25°C. The test subject walks 2.5 m, which according to his height and cadence represents a complete cycle of the gait. For the experiment, 20 tests were carried out with the same individual, the data obtained during the experiment was plotted using MATLAB® 2018 software. In Figure 6A, graphs plotted in the software are observed with the data of the voltage variation obtained in each sensor during a gait cycle, without digitally processing them. The normalization equation given in (1) was applied to the data obtained, the results from the experiment were averaged for each of the sensors, on the horizontal axis the time is indicated in seconds, while the force value in Newtons is indicated on the vertical axis, a horizontal axis was added referring to the percentage of the gait cycle.

From the Figure 6A, the contact time of each part of the foot was determined, considering that the forces are only present during the stance phase of the gait cycle, because at this period there is a contact between the ground and the foot. In the graph corresponding to sensor 1, the stage called **first support** is being carried out, where the heel contacts to the ground supporting most of the body's weight for an instant, it can be seen how the force grows suddenly and then descends gradually to start the next stages when the foot contacts the ground. In the subsequent graphs corresponding to the sensors located in the anterior part of the foot (2, 3, 4), the part of the phase called **plantar support** is carried out, this is observed when the force in the three remaining sensors changes gradually, until reaching its maximum point (**terminal support**) when the heel is completely separated from the ground, finally, it is observed how instantly the force decreases, until it reaches a minimum value (**take-off** phase), ensuring that the foot has not contact with the ground starting the swing phase of the gait cycle.

The average of the GRF was obtained in (2), using the insole and the mathematical model for the sensor given in (1),


(2) $$\begin{array}{r} GRF_{v}=\frac{1}{p}\;\overset{p}{\underset{i=1}{\sum }}F_{i}\left(t\right) \end{array}$$

Where *GRF*_*v*_ are average of the vertical ground reaction forces, *p* is the number of samples measured by the DAS, and *F*_*i*_ are the force value of each FSR sensor. Using (2) the graph in Figure 6B was obtained, where the variation of the forces depending on the percentage of the stance phase of the gait cycle. By comparing the shape and data obtained from the graph with the data reported in the literature (Nordin and Frankel, 2013) the correct functioning of the instrumented insole was verified.

The instrumented insole presents great usability and portability, it can communicate with different digital devices via Bluetooth, allowing it to be an independent system that can be used as a diagnostic tool for walking pathologies for the analysis of gait cycle measuring the GFR obtained. An additional application is described in the development of the semiactive knee orthosis presented in this article, sending GRF data to the control system to regulate the behavior of the MR damper and therefore of the orthosis.

In this way, it was verified that the information sent by the DAS are required by the control system to regulate the damping of the orthosis based on controlling the supply current of the MR actuator.

### MR Actuator Control System

**Figure 7:**
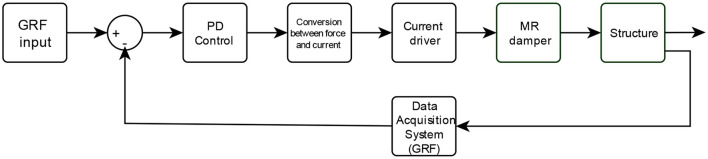
Block diagram of the designed control system.

For the design of the control system is necessary to obtain a model that describes the function of each component of the orthosis, explaining the interconnection between the DAS, the controller designed and the change of damping force on the MR actuator (Weber, 2015). The control problem is to regulate the damping force of the semiactive damper from GRF measured by the DAS to protect the knee from forces to which it is subjected during walking. The plantar pressures regulate the current flow that reaches the MR damper, consequently, depending on this variation, the damping force of the actuator is increased or decreased, so we can say that the value of the damping force is directly related to the value of ground reaction forces and therefore depends on the characteristics of the gait cycle of each user, in Figure 7 the block diagram of the designed control system is presented.

#### SALLO Model

**Figure 8:**
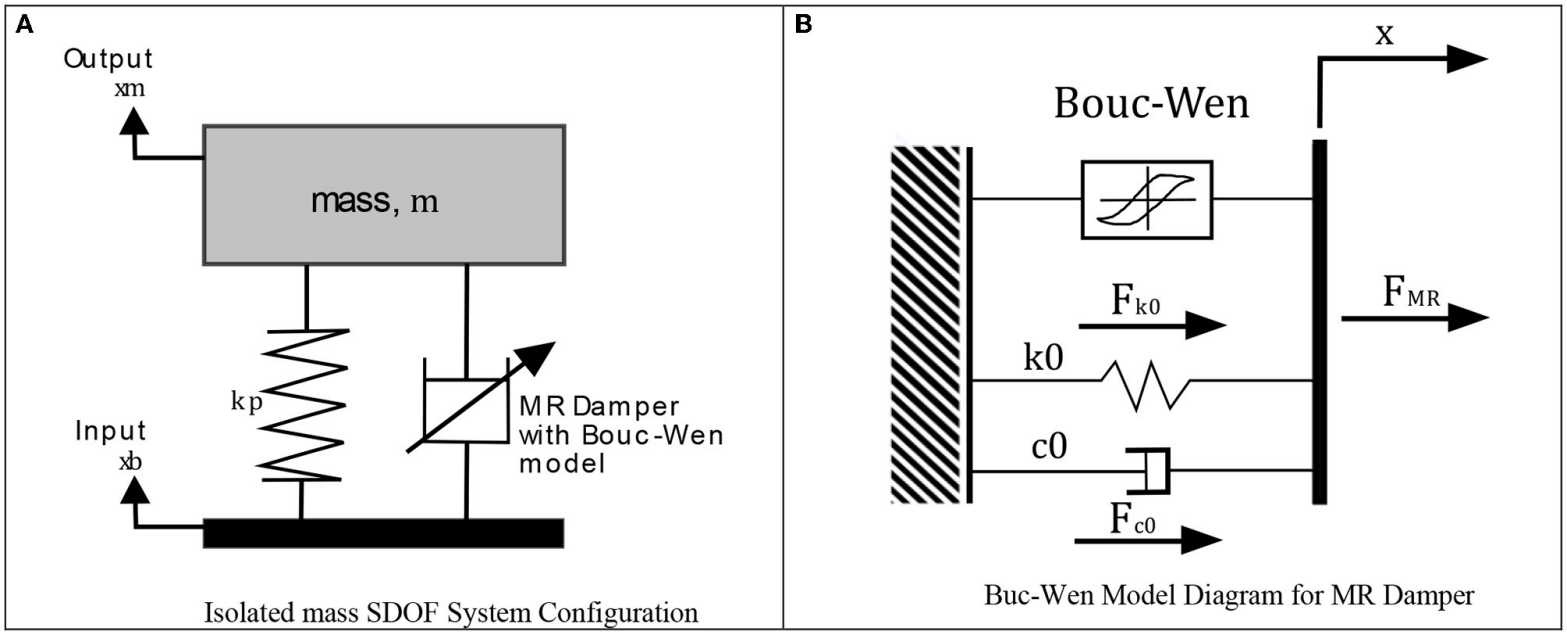
Diagrams for the configuration of semiactive orthosis. **(A)** Isolated mass SDOF system configuration. **(B)** Buc-Wen model diagram for MR damper.

This knee orthosis prototype incorporating an MR damper can be modeled according to Ahmadkhanlou (2008) as a Single Degree of Freedom (SDOF) system, considering the hysteresis behavior of the MR damper adding a spring mass system in Figure 8A, the model reacts to an input signal, which in this case corresponds to the movements generated in the knee during the gait cycle, or when performing daily activities.

The system presented in **Figure 12**, is modeled through the analysis of forces that interact in each component, in such a way that Equation (**3**) is obtained.


(3) $$\begin{array}{r} F_{r}=\;-F_{k_{p}}-F_{MR} \end{array}$$

Where *F*_*r*_ is the resultant force of the system, and *F*_*kp*_ is the force performed by the leg muscles and *F*_*MR*_ is the damping force of the MR actuator (4), the dynamic model given in (5) is obtained,


(4) $$\begin{array}{rcl} m{ẍ}_{m} & = & \;-k_{p}\left(x_{m}-x_{b}\right)-F_{MR} \end{array}$$

(5) $$\begin{array}{rcl} {\ddot{x}}_{m}\left(t\right) & = & \;-\frac{k_{p}}{m}\left(x_{m}\left(t\right)-x_{b}\left(t\right)\right)-\frac{F_{MR}\left(t\right)}{m} \end{array}$$

Where ẍ_*m*_ is the acceleration of the system, *x*_*m*_ and *x*_*b*_ are the positions of the system before the input signal, and *F*_*MR*_ is considered as the force due to the behavior of the damper.

#### MR Damper Model

Inside the SDOF model a MR actuator with a rheological fluid is included. A fluid with this property can modify its mechanical properties according to the magnetic field applied to it, which oppose a movement causing the activation of the piston when exceeds certain threshold. Specifically, magnetorheological fluids contain microscopic particles with some ferromagnetic material, when these particles are exposed to a magnetic field, this fluid changes its resistance, converting the behavior of the fluid to a semi-solid material and in the absence of the magnetic field (Russo and Terzo, 2011), it returns to being a liquid. These ferromagnetic particles orient themselves when there is interaction with an electromagnetic field. The control of these actuators is complex due to their behavior with hysteresis.

In the design of this orthosis, the MR RD-8040-1 actuator from LORD Corporation^®^ was selected, various mathematical models have been reported in the literature to identify the behavior of this device, including the model developed in Wen (1976), where the behavior of the damper as a classic spring and damper system is considered adding the hysteresis action (Figure 8B).

The parametric Bouc-Wen model is used because its present low computation cost, and simplest implementation on embedded systems (Sapinski and Filús, 2003). According to the diagram in Figure 8, the damping force of the MR damper is described in (6)


(6) $$\begin{array}{r} F_{MR}=F_{k0}+F_{c0}+h\left(z\right) \end{array}$$

Where the term *F*_*MR*_ presents the summatory forces, due de the spring force *F*_*k*0_, and the damping force *F*_*c*0_ generated by the displacement of the actuator piston, whereas *h*(*z*) is a function that depends on the displacement of *x*, where *z* is an evolutionary variable that contains the characteristics of the behavior with hysteresis of the system, the dynamic equations of the MR actuator are presented in (7) and (8),


(7) $$\begin{array}{r} F_{MR}\;=\;c_{0}ẋ+\;k_{0}\left(x-x_{0}\right)\;+\text{ α}z \end{array}$$

(8) $$\begin{array}{r} \overset{∙}{z}=\;-\gamma |x|z|z{|}^{n-1}-\beta \overset{∙}{x}|z{|}^{n}+A\overset{∙}{x} \end{array}$$

Where *c*_0_ represents the viscous damping coefficient; *k*_0_ the stiffness coefficient due the accumulator; *x*_0_ is the initial displacement; α presents the ratio of the yield value; *z* is the variable displacement with hysteresis; the parameters γ, β, and *A* control the discharge linearity; the parameter *n* is the coefficient of smoothness of the curve.

To define the parameters of the previous equations in Priya and Gopalakrishnan (2016), an experiment was developed specifically for the RD-8040-1 damper, using the Bouc-Wen model and the equations representing the variation of the intensity of current that flows toward the coil of this device, the variables α (9), *c*_0_ (10), and *k*_0_ (11) were modified.


(9) $$\begin{array}{r} \alpha \left(u\right)=\;\alpha_{a}+\alpha_{b}u \end{array}$$

(10) $$\begin{array}{r} c_{0}\left(u\right)=\;{c_{0}}_{a}+{c_{0}}_{b}u \end{array}$$

(11) $$\begin{array}{r} k_{0}\left(u\right)=\;{k_{0}}_{a}+{k_{0}}_{b}u \end{array}$$

Where *u* is obtained from the input current *I* (12).


(12) $$\begin{array}{r} \overset{∙}{u}=\;-\eta \left(u-I\right) \end{array}$$

Where η is a variable related to the response of the actuator depending of current.

**Figure 9:**
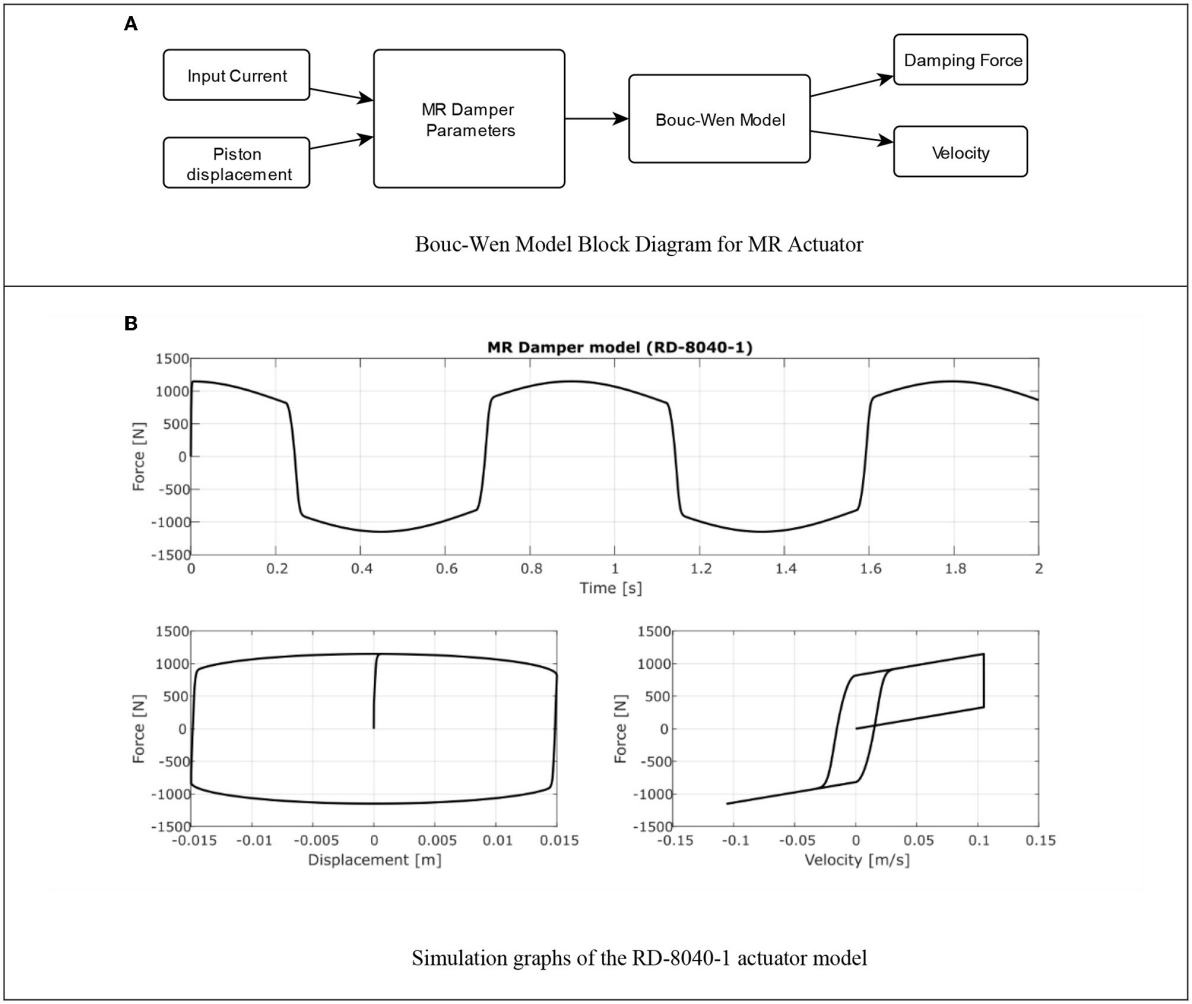
MR Damper simulation. **(A)** Bouc-Wen model block diagram for MR actuator. **(B)** Simulation graphs of the RD-8040-1 actuator model.

Figure 9A is observed the block diagram where is implemented the simulation of the MR actuator behavior, using MATLAB^®^ R2018 and its SIMULINK^®^ toolbox, to identify the damping force of the semiactive damper as a function of the variation of current.

To simulate the piston displacement of the damper, a sinusoidal signal with an amplitude of 0.015 m at a frequency of 3 Hz (like walk at 1.5 *ms*^−1^), and a current value of 1 A, was used as the desired piston displacement. The simulation time is 2 s, resulting in two cycles of the input signal. In Figure 9B, the simulation results are presented, in the upper graph the force against time is observed, the force reaches a peak value of ±1,149 N. In the lower right graph, the relation between the force and the piston displacement of the damper is observed and finally in the lower left figure the relation between the damping force and the response speed of the actuator is observed. The velocity was obtained from the numerical derivative of the displacement function, in this simulation is not considered the accumulator force of the MR damper.

With the results of the simulation, it is verified that the Bouc-Wen model adequately represents the behavior of the RD-8040-1 damper (Arias-Montiel et al., 2015), considering its response with hysteresis, as indicated by the manufacturer. The high response speed was observed before the change of the force value, it was also identified that with a current of 1 A damping forces >1,000 N are obtained, therefore its adequate performance is confirmed using a power supply of low amperage, which is one of the characteristics remarkable in this device and which is a requirement for the design of the proposed orthosis.

**Figure 10:**
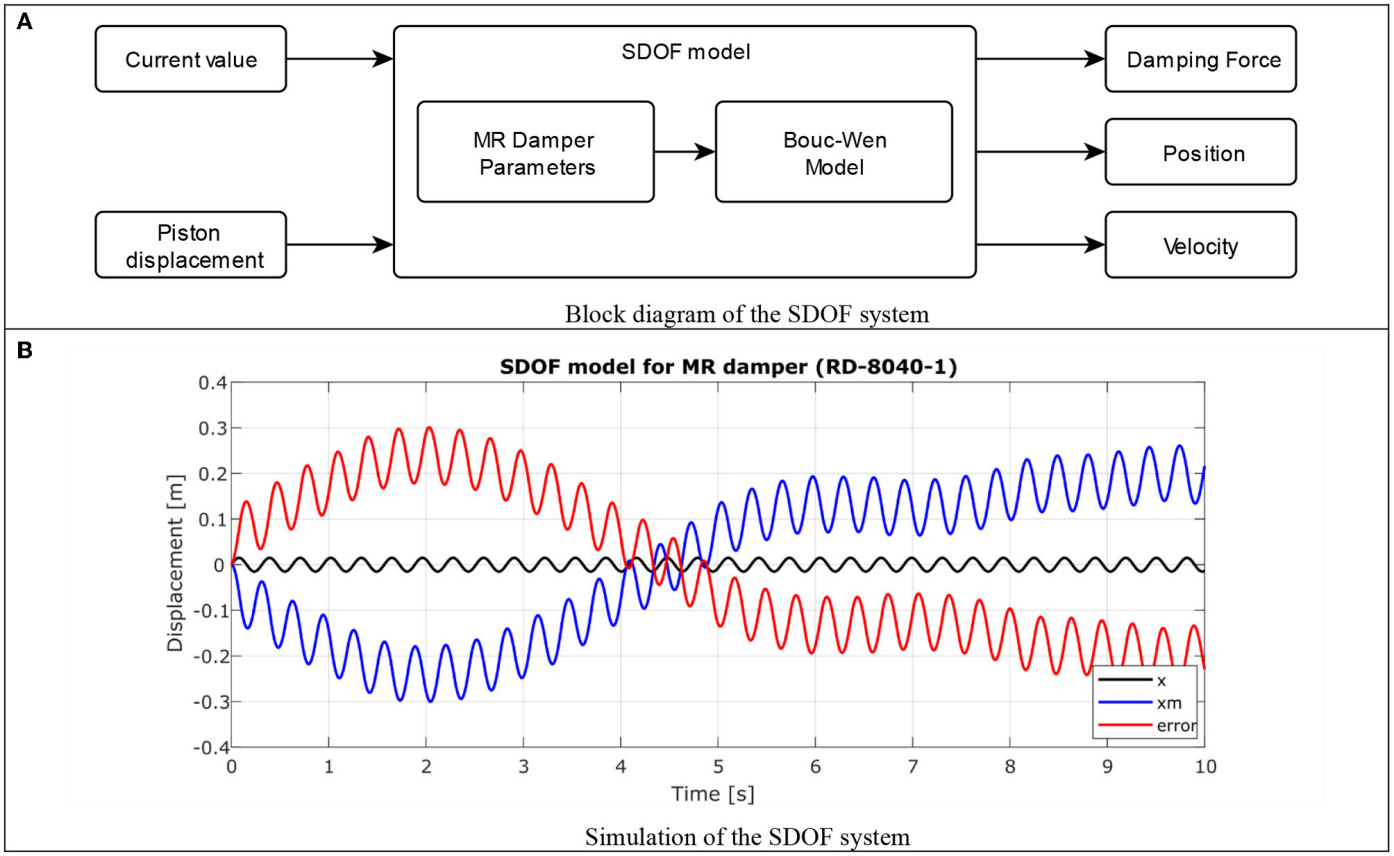
Simulation of single degree of freedom system. **(A)** Block diagram of the SDOF system. **(B)** Simulation of the SDOF system.

Using the MR damper model and Equation (**7**), a block diagram is generated in the SIMULINK^®^ toolbox of the MATLAB^®^ 2018b software to simulate the behavior of the SDOF, including the Bouc-Wen model. *m* = 80 kg and *kv* = 22*Nm*^−1^ representing the mass and stiffness of the leg muscles identified in Kuitunen et al. (2011), were used as values in this experiment, this diagram is shown in Figure 10A.

In Figure 10B, three signals are presented from the simulation of the SDOF model and integrating the Bouc-Wen model for MR actuator without damping control. The signal in black *x* represents the input signal of the desired displacement of the piston, in this case a sinusoidal wave with a frequency of 3 Hz (like walk at 1.5 *ms*^−1^) and an amplitude of 0.015 m is used, simulating small variation on the stem. The signal in blue color shows *x*_*m*_ which is the response of the system's displacement according to the input signal, when observing the signal, it is verified that at no time the system is stabilized. In red color the error generated between *x* and *x*_*m*_ is observed.

In such a way that, if it is desired to regulate the damping of the system, it is necessary to establish a control strategy to achieve that the error tends to zero.

#### Proportional Derivative Controller

**Figure 11:**
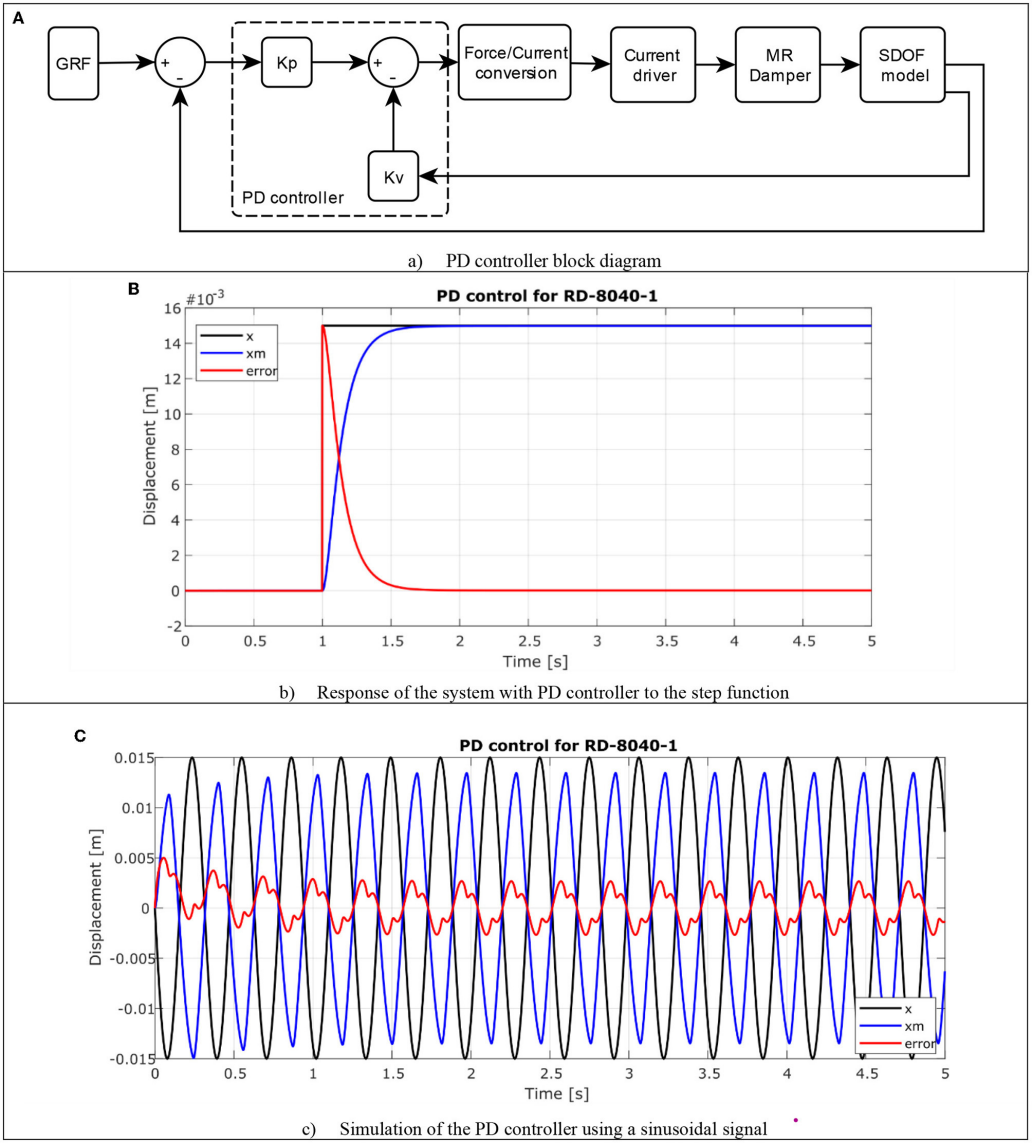
Proportional-derivative controller. **(A)** PD controller block diagram. **(B)** Response of the system with PD controller to the step function. **(C)** Simulation of the PD controller using a sinusoidal signal.

The proposed objective for the control is to regulate the current required by the MR actuator to modify its damping force, for which a proportional derivative PD controller was used. The controller structure is presented in Equation (13), it is composed of two tuning variables *K*_*p*_ and *K*_*v*_. Where *K*_*p*_ is the proportional gain of the control system, its main characteristic is based in that the position error is directly proportional, $\tilde{q}$; while *K*_*v*_ is the derivative gain which has a damping effect on joint velocity $\overset{∙}{q}$. The position error, $\tilde{q}$ is the difference between the desired position value and the obtained value $\tilde{q}\left(t\right)=\;q_{d}\left(t\right)-q\left(t\right)$. In Figure 11A, the block diagram is observed applying this type of controller to the system.


(13) $$\begin{array}{r} F_{PD}=\;K_{p}\tilde{q}\left(t\right)-K_{v}\overset{∙}{q}\left(t\right) \end{array}$$

Considering Equation (13), this controller is added to the simulation of the behavior of integrated system carried out in the SIMULINK^®^ toolbox of the MATLAB^®^ 2018b software (Figure 11A).

To verify the behavior of the PD controller, the input signal was initially modified by a step function, with the same amplitude of 0.015 m in Figure 11B, the response of the system with the controller is observed. The response obtained by the controller is the desired one, reaching the 15 mm of displacement by the stem in a time of ~0.5 s, with an error that tends to cero, so the controller meets its objective.

For the second simulation, the signal described in the first simulation without control was used, a sinusoidal wave with a frequency of 3 Hz (like walk at 1.5 *ms*^−1^) and an amplitude of 0.015 m in Figure 11C, the results are observed. The signal displacement *x* in black is followed by the current displacement *x*_*m*_ in blue with a signal delay due to the behavior of the damper, and the absolute error in red is around 5%. By applying the control strategy, the system response is stable according to the input signal with a minimum absolute error, in this way it is verified that the simulation control system does regulate the damping force of the semiactive actuator of the orthosis.

The designed controller adapts to the needs of the proposed system, considering the behavior of the MR actuator and the complexity of the integrated system, improving the damper's response according to the simulation executed. The simple structure of this controller allows its implementation on an embedded system where the computing capacity is relatively low.

### Structure of the Prototype Knee Orthosis

For the design of the orthosis structure, different factors were taken into consideration, user requirements, easy manufacturing, accessible cost, etc. Additionally, the following specifications were considered:

1. The structure must support the MR actuator, whose mass is 800 g.
2. It should be ergonomically adapted to the contours of the leg, for a proper fit. The anthropometric measurements of the human body from the study presented in Ávila Chaurand et al. (2007) were considered, where the length of the lower limb for a man within a range of 20–40 years is 0.54 m for the thigh and 0.45 m for the leg.

**Figure 12:**
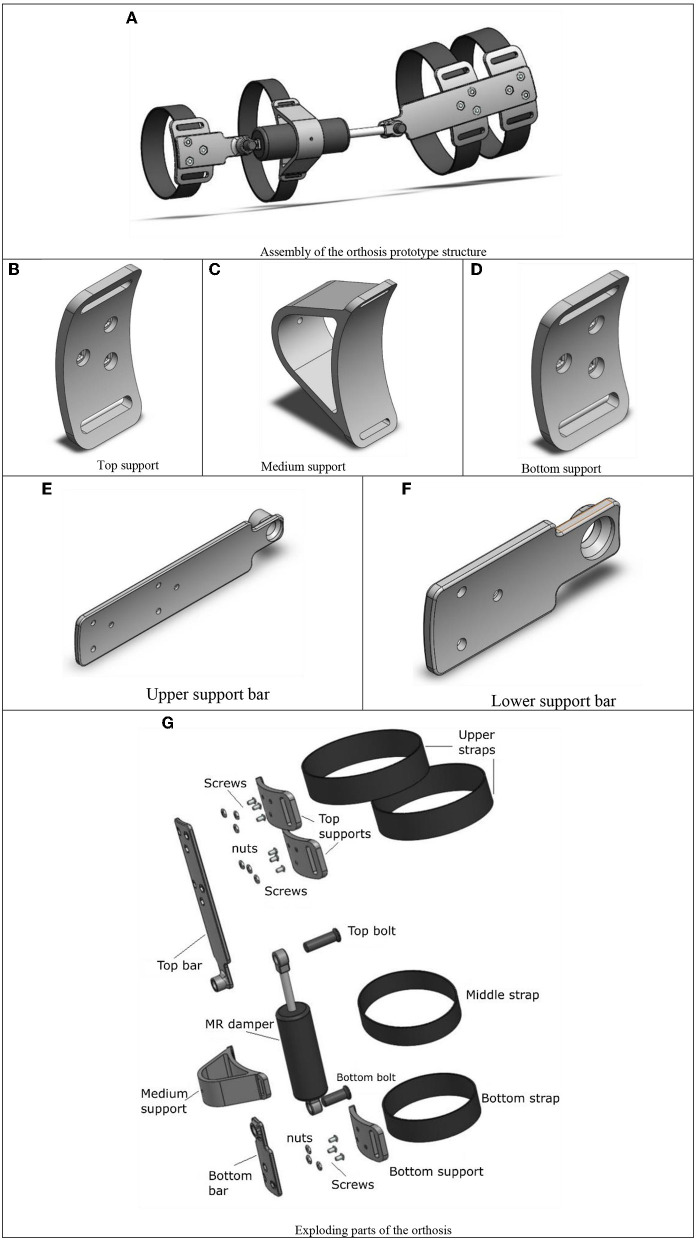
CAD model of the semiactive orthosis. **(A)** Assembly of the orthosis prototype structure. **(B)** Top support. **(C)** Medium support. **(D)** Bottom support. **(E)** Upper support bar. **(F)** Lower support bar. **(G)** Exploding parts of the orthosis.

The structure of the knee orthosis that incorporates a semiactive actuator was designed in the SolidWorks^®^ 2016 CAD software, show in Figure 12A.

Three horizontal curved supports were designed which adapt to the morphology of the leg, in Figure 12B the upper support that is placed on the thigh is observed, the middle support observed (Figure 12C) is placed on the cylinder of the MR damper to give stability and location, and the third support, lower support (Figure 12D) is placed on the leg.

Two vertical support bars were designed, which aim to link up the two extremes of the MR actuator. The longest support (Figure 12E), links the upper part of the system, this bar is placed on the thigh and the extreme of the piston. The lower bar (Figure 12F), links the base of the actuator and positions it on the leg to secure the location of the device.

In Figure 12G, each of the components of the orthosis structure is observed from an exploded, including four Velcro bands whose function is to adjust the orthosis to the user's leg.

To guarantee the safety of the user, several simulation experiments were carried out with the finite element method applied to each piece of the structure and its assembly, to determine if its structural components support the loads to which they are going to be bring under. These simulations allow to determine by the maximum displacements and von Mises stresses identify if the structure fails under a certain load.

The ANSYS^®^ software in its 2019R version was used to simulate the conditions and considering the mechanical properties of the plastic material in which the structure was manufactured, this is a polymer named Polylactic Acid or PLA, which yields high mechanical properties in terms of traction, it also has biodegradable properties, while the MR actuator material is stainless steels 304. Importing the CAD model of the mechanism, into the ANSYS interface, adding the properties of the materials already mentioned and using 3D structural elements SOLID186, the meshing selected for each element applying a load of 800 N on the piston of the damper was performed, simulating the weight of a person of 80 kg, this point was selected because of the greatest stress supported, in addition, movement restrictions are placed in the locations where the screws were placed.

**Figure 13:**
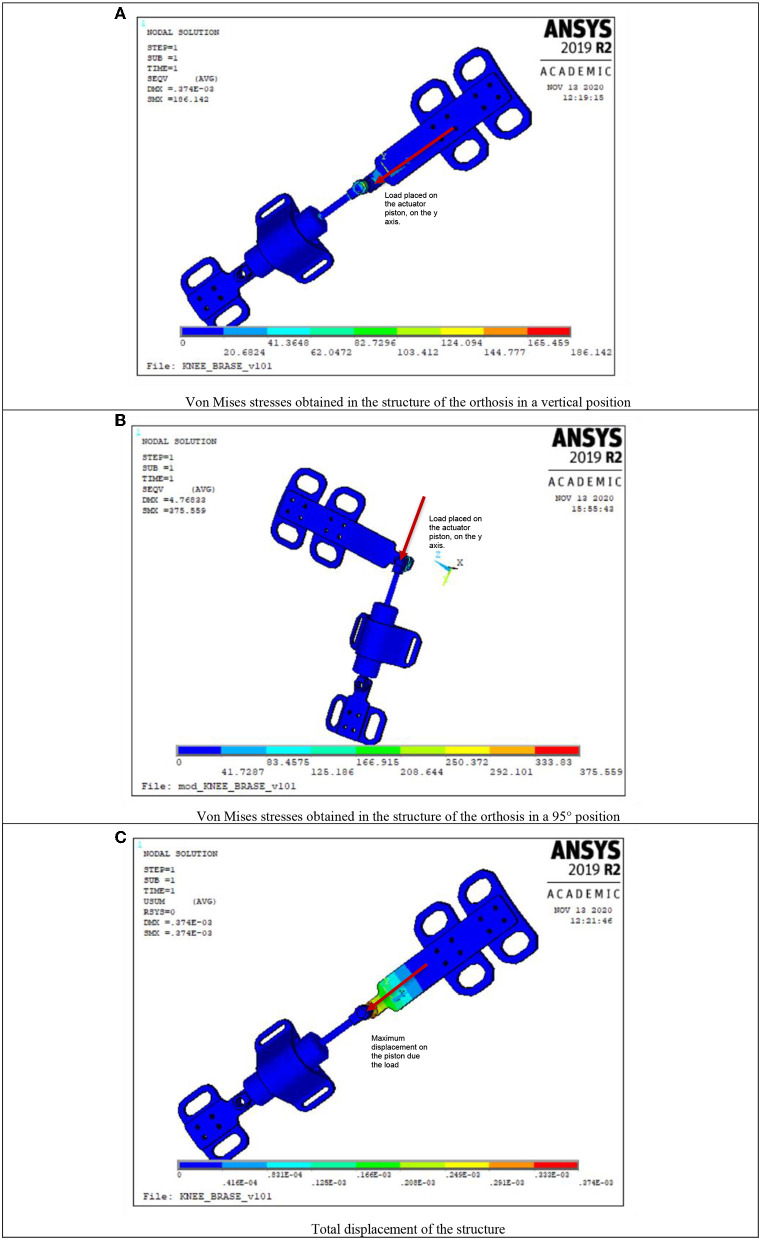
Stress analysis on ANSYS^®^. **(A)** Von Mises stresses obtained in the structure of the orthosis in a vertical position. **(B)** Von Mises stresses obtained in the structure of the orthosis in a 95° position. **(C)** Total displacement of the structure.

Several analyzes of the structure were carried out in different positions, especially the critical cases where the assembly supports the greatest stress. In Figure 13A, the results of the von Mises stresses generated during the simulation with the assembly at a 180° angle (simulating when the leg is fully extended) were presented. Similarly, in Figure 13B, the results of von Mises stresses with assembly of 95° (representing the knee flexion movement) were shown.

According to the results observed in Figures 13A,B, the von Mises stresses for the 180° angle are 186 N in the vertical position, while for the 95° assembly this value is 375 N, these values are under the yield stress of PLA which is 2.9 and 193 GPa for the stainless steel, ensuring that the operation of the structural components will not be compromised by this load. In Figure 13C, a maximum displacement of the structure is observed with a load of 800 N, which is only 0.37 mm.

**Figure 14:**
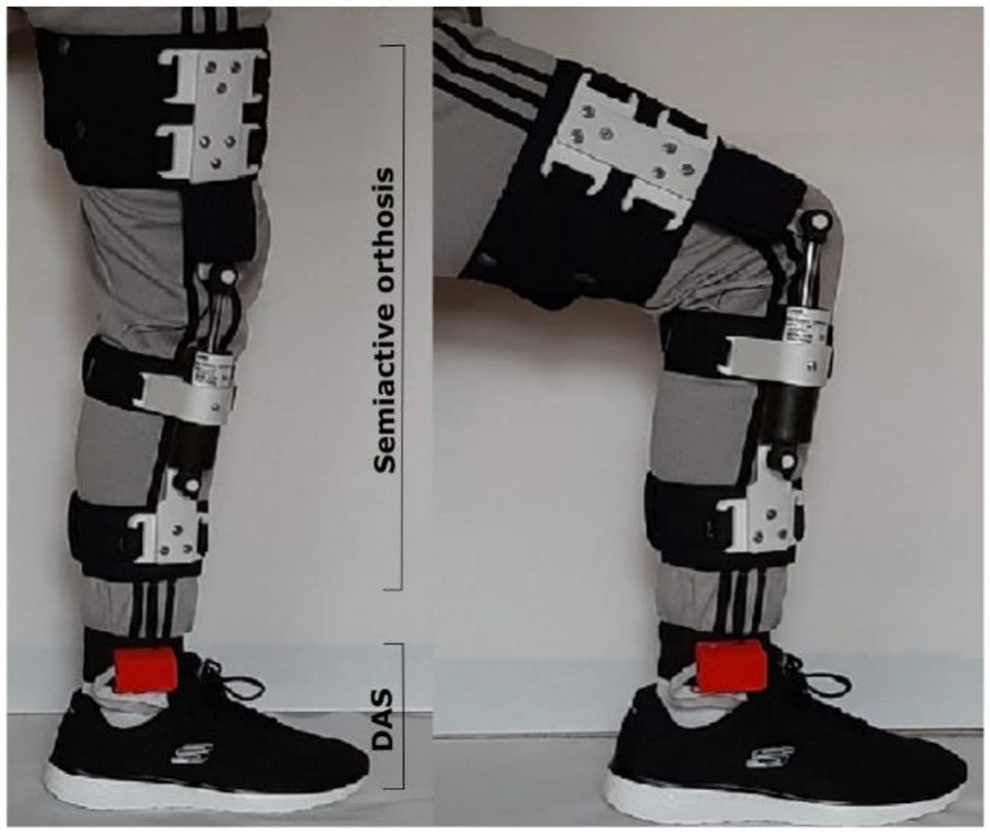
3D printed orthosis structure placed on the leg.

The simulation results showed that the layout of the elements can support the efforts generated by an 80 Kg individual, considering that the manufacturing material is PLA. Once the design was validated, 3D printing was used to manufacture the components of the structure. Additive manufacturing is one of the processes that has revolutionized the visualization of prototypes, whose main peculiarity is to superimpose layers of material, and then take the desired shape, minimizing the time of processing. With the CAD models presented in Figure 12G each component was manufactured, using a CREALITY brand machine in its ENDER 3 model. The characteristics with which the pieces of the orthosis structure were printed, selecting a material density of 20% it is not a solid piece, since there are spaces between the layers of material, making the pieces lighter, but with high tensile strength. The measurements correspond to a scale 1 to 1, achieving a tolerance of 0.1 mm, considering the thermal expansion of the material. The printing was made following a tetrahedron pattern in a vertical direction, this due to the forces that interact in the system, avoiding a possible fracture within the components. In Figure 14, the printed parts and the assembly of the structure are observed from the generated model. The bands that adjust the location on the lower limb were manufactured with cotton cloth to avoid irritation on the skin, adding Velcro strips to adjust to the size of the user's leg, to link up the components standard screws was used.

## Results

To validate the operation of the orthosis, it was carried out experiments with healthy users using the smart insole to measure the GRF and this data is sent to the control system to regulate the damping force to the MR actuator. Subsequently, an experiment was designed and executed to verify the behavior of the SALLO system in a healthy user, for this the GRF was measured in two cases; the first when the orthosis is utilized and the second the orthosis is not being utilized.

### Experimental Validation of the Insole

**Figure 15:**
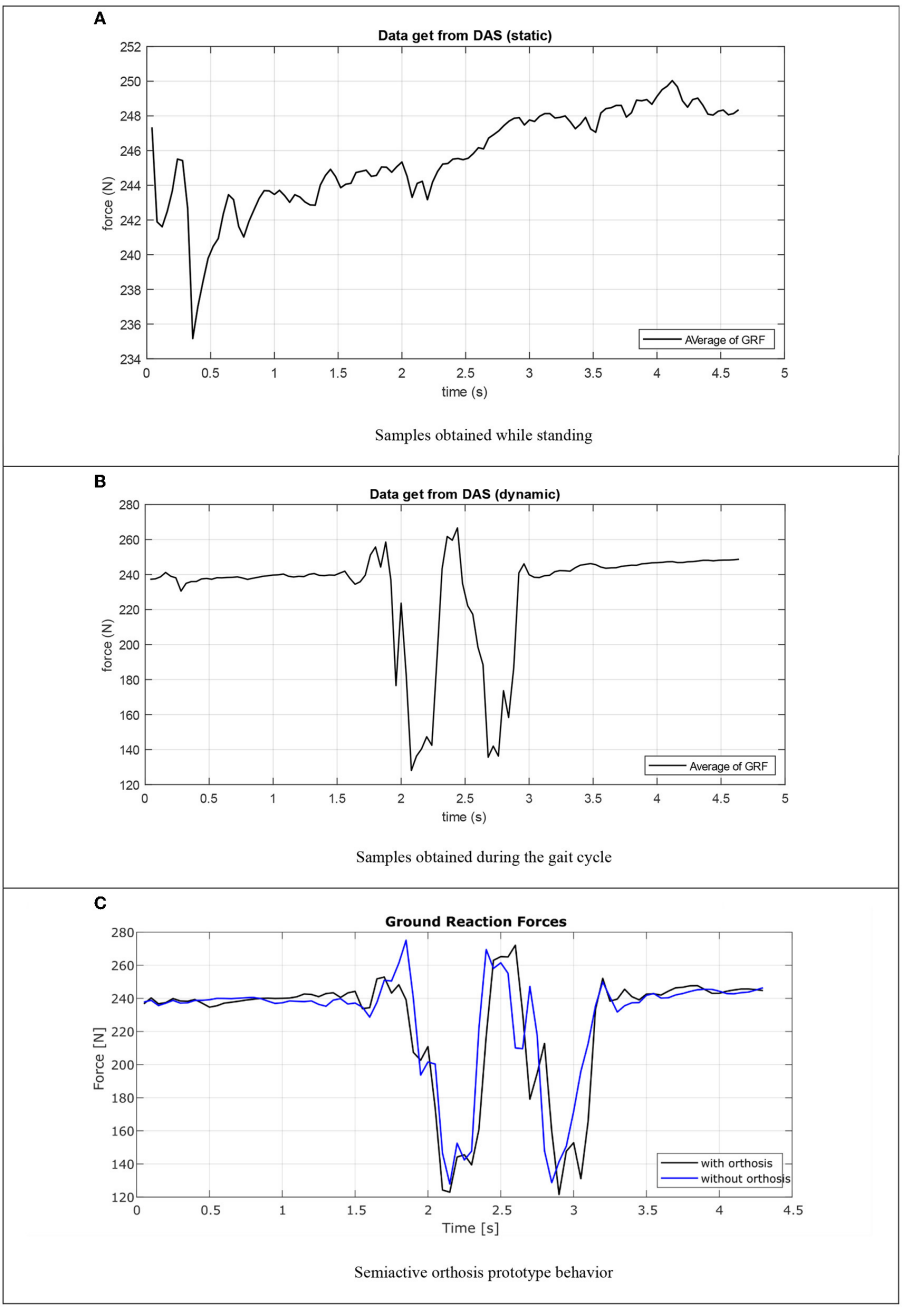
Semi-active orthosis evaluation. **(A)** Samples obtained while standing. **(B)** Samples obtained during the gait cycle. **(C)** Semiactive orthosis prototype behavior.

To verify the behavior of the DAS integrated to the orthosis, an experiment was performed to determine the efficiency of this subsystem, the first part of this experiment contemplate a static analysis where the user stands for 4.5 s, consider that the user is 1.8 m tall and weighs 80 Kg. In Figure 15A, the graphs obtained using the MATLAB^®^ 2018b software are observed after completing a series of samples, the data is plotted by averaging the values of all measurements, in black showing the ground reaction forces when standing, where the vertical axis corresponds to the forces in Newtons and the horizontal axis to time, the observed variations depend directly on the position of the user, the data indicates that the system in static mode tends to a value between 242 and 255 N after the user stabilization. Table 1 showed the relationship between the three values of interest, the GRF measured by the data acquisition system, the value of the driving current, and the damping force on the semiactive actuator. These values are strongly related if the ground reaction forces increase the other two values also increase, in summary, the damping force value changes when the GRF changes depending completely on the characteristics of the human gait cycle.

**Table 1:** Relation between GRF, current, and damping force.

**GRF (*N*)**	**Current (A)**	**Damping force (*N*)**
177.1405	0.16	255
185.9098	0.20	297
194.6792	0.24	340
203.4478	0.28	382
212.2178	0.32	425
220.9871	0.35	467
229.7565	0.39	510
238.5258	0.42	553
247.2951	0.46	595
256.0644	0.51	638
264.8338	0.54	680
273.6031	0.57	723
282.3724	0.61	765
291.1417	0.65	808
299.9111	0.69	851
308.6804	0.74	893
309.4520	0.78	936
318.2129	0.83	978
327.6972	0.88	1,021
336.4512	0.92	1,063
345.3688	0.96	1,106
354.7845	1	1,149

The second experiment was carried out with the same test subject, but now the experiment consists of standing for 2 s and then taking a step and finally standing again for 2 s, the force values on this time gets averaged and plotted in black as seen in Figure 15B with the same axes. As in the static mode, once the user has stopped his movement, the measured forces are close to the mean value of 250 N. Considering the maximum and minimum values that the data acquisition system obtain from the measurement of ground reaction forces, a range of force values generated during the gait cycle ranging from 170 to 350 N was determined, in accordance with the information reported in the literature. It is observed that the response curve in both cases is also like the curves obtained in different works reported in the literature, where the systems use a greater number of sensors in the insole and longer processing time than that achieved with the DAS proposed in this work.

### Validation of the SALLO Operation

The validation of the orthosis operation was carried out by setting up an experiment with a healthy individual weighing 80 kg and 1.8 m tall, in a closed room at room temperature. Where the individual walk distance of 2 m, which represents a complete gait cycle. The experiment begins by holding the standing position for a couple of seconds, then a full step is taken and finally it is returned to the initial standing position. The experiment was first performed on the user without wearing the orthosis and then the experiment was repeated using the orthosis.

In Figure 15C, GRF measurement in the individual's foot obtained with DAS is observed, the blue line represents the obtaining of data when the orthosis prototype is not used, while in the black line the GRF is plotted when the individual is wearing the semiactive orthosis prototype, the vertical axis represents the force evaluated in Newtons, while the horizontal axis measures the time in seconds.

The data from the DAS show that there is a decrease in the ground reaction forces when the SALLO prototype is used, compared to the data obtained when it is not used, the decrease is around 12%. This is because the orthosis is absorbing the forces when the individual uses the design prototype despite not suffering any injuries. It can be verified that the orthotic device fulfills the function of controlling the damping force on the semiactive actuator when the forces act on the knee during the gait cycle, as is showed in Figure 15C. It can be observed on the peaks where the force presents high values, for example, when the heel and forefoot contacts with the ground are made, in the graph without orthosis these peaks are higher and in the graph that represents the results when the orthosis the peaks are less pronounced, and the signal is softer. This implies that the MR actuator damping control does offer support to the knee, preventing it from being overstressed when performing daily activities such as walking.

Similarly, taking measurements on the controller signal, it was determined that the time delay that takes to modify current value for the MR actuator is <100 ms, considering that the measurements made during the gait cycle, where a step lasts between 1.5 and 2 s at a speed of 1.5 *ms*^−1^, so there are 15–20 changes in the value of the current which are reflected in the damping force generated by the RD-8040-1 actuator.

## Conclusions

According to the results obtained by experimentation, it was possible to design a functional prototype of a semiactive knee orthosis, which combines a structure that supports and adapts to the morphology of the leg with an MR actuator whose main function is to mitigate the forces supported by the knee increasing or decreasing its damping force by modifying the magnetic field inside the actuator, regulating the current flow according to a portable system for measuring the GRF of the user's foot embedded in an instrumented insole that goes inside the user's shoe. The system presents competitive advantages when comparing its operation with the prototypes presented in the literature, since sensorimotor effects are considered in the design from GRF data sent to the system allowing the regulation of the feed current to the MR actuator and consequently modifying its damping force depending on the period of the support phase of the gait cycle that is being executed. As a result, a decrease in the reaction forces on the knee is observed, especially during the highest-pressure peaks, expressly during the landing on the ground of the heel and forefoot. The advantages are centered on the reduced size of the system, its autonomy of up to 3 h, a better resolution in the data acquisition when using a 12-bit ADC and the possibility of implementing the PD control in a reduced system. The software architecture developed allows modifications to improve the processing of the signal obtained from the sensors, such as adding digital filters without modifying the hardware. Additionally, a 12% reduction on the GRF, when the prototype is used, is showed, we can infer that the results can be replicating on a test subject with an abnormal gait, increasing their performance when walking.

## Data Availability Statement

The original contributions presented in the study are included in the article/supplementary material, further inquiries can be directed to the corresponding author/s.

## Author Contributions

DA-R, PN-S, and LC-R conceived, designed, performed the experiments, analyzed the data, and wrote the paper. All authors contributed to the article and approved the submitted version.

## Funding

The present research has been partially financed by SIP project: 20221843, and by Consejo Nacional de Ciencia y Tecnología, CONACYT.

## Conflict of Interest

The authors declare that the research was conducted in the absence of any commercial or financial relationships that could be construed as a potential conflict of interest.

## Publisher's Note

All claims expressed in this article are solely those of the authors and do not necessarily represent those of their affiliated organizations, or those of the publisher, the editors and the reviewers. Any product that may be evaluated in this article, or claim that may be made by its manufacturer, is not guaranteed or endorsed by the publisher.
